# A systematic review and synthesis of the strengths and limitations of measuring malaria mortality through verbal autopsy

**DOI:** 10.1186/s12936-017-2071-x

**Published:** 2017-10-23

**Authors:** Samantha Herrera, Yeetey Enuameh, George Adjei, Kenneth Ayuurebobi Ae-Ngibise, Kwaku Poku Asante, Osman Sankoh, Seth Owusu-Agyei, Yazoume Yé

**Affiliations:** 1 MEASURE Evaluation, ICF, 530 Gaither Road, Suite 500, Rockville, MD 20850 USA; 2 Kintampo Health Research Centre, Kintampo, Ghana; 3 School of Public Health, Kwame Nkrumah University of Science and Technology, Kumasi, Ghana; 4 INDEPTH Network, 38 & 40 Mensah Wood Street, East Legon, Accra, Ghana; 5 School of Public Health, Faculty of Health Sciences, University of the Witwatersrand, Johannesburg, South Africa; 6 Department of Mathematics and Statistics, Njala University, Njala, Sierra Leone; 7 Institute of Health Research, University of Health and Allied Sciences, Ho, Ghana

**Keywords:** Malaria mortality, Verbal autopsy, Cause-specific mortality

## Abstract

**Background:**

Lack of valid and reliable data on malaria deaths continues to be a problem that plagues the global health community. To address this gap, the verbal autopsy (VA) method was developed to ascertain cause of death at the population level. Despite the adoption and wide use of VA, there are many recognized limitations of VA tools and methods, especially for measuring malaria mortality. This study synthesizes the strengths and limitations of existing VA tools and methods for measuring malaria mortality (MM) in low- and middle-income countries through a systematic literature review.

**Methods:**

The authors searched PubMed, Cochrane Library, Popline, WHOLIS, Google Scholar, and INDEPTH Network Health and Demographic Surveillance System sites’ websites from 1 January 1990 to 15 January 2016 for articles and reports on MM measurement through VA. Inclusion criteria: article presented results from a VA study where malaria was a cause of death; article discussed limitations/challenges related to measurement of MM through VA. Two authors independently searched the databases and websites and conducted a synthesis of articles using a standard matrix.

**Results:**

The authors identified 828 publications; 88 were included in the final review. Most publications were VA studies; others were systematic reviews discussing VA tools or methods; editorials or commentaries; and studies using VA data to develop MM estimates. The main limitation were low sensitivity and specificity of VA tools for measuring MM. Other limitations included lack of standardized VA tools and methods, lack of a ‘true’ gold standard to assess accuracy of VA malaria mortality.

**Conclusions:**

Existing VA tools and methods for measuring MM have limitations. Given the need for data to measure progress toward the World Health Organization’s *Global Technical Strategy for Malaria 2016*–*2030* goals, the malaria community should define strategies for improving MM estimates, including exploring whether VA tools and methods could be further improved. Longer term strategies should focus on improving countries’ vital registration systems for more robust and timely cause of death data.

**Electronic supplementary material:**

The online version of this article (doi:10.1186/s12936-017-2071-x) contains supplementary material, which is available to authorized users.

## Background

Lack of valid and reliable data on malaria deaths, especially in endemic countries which house the greatest burden, continues to be a problem that plagues the global health community. In light of the recent adoption of the sustainable development goals (SDGs) and the World Health Organization’s *Global Technical Strategy for Malaria 2016*–*2030* [1, 2], it presents a challenge to the community on how best to capture malaria mortality data to assess progress on the ambitious goals and targets set within these agendas. Accurate data on malaria mortality at the national and subnational levels is essential for effective policy-making and programme planning; it will also be critical for countries as they move toward low transmission or pre-elimination status to monitor and evaluate progress and to adapt programmatic strategies as the burden declines.

Measuring malaria-specific mortality at the population level is challenging due to the lack of complete vital registration systems in most low- and middle-income countries, the difficulty in clinical assessment of malaria, and the fact that most malaria deaths occur outside of the formal health care system [3–5]. To address this gap, the verbal autopsy (VA) method was developed to ascertain cause of death (COD) at the population level. Global and country specific data on malaria mortality is thus largely derived from incomplete vital registration data and supplemented with VA data, and in some instances inpatient mortality data, to produce estimates of the number of malaria deaths.

Verbal autopsy consists of an interview conducted with a family member or an individual familiar with the deceased using a structured questionnaire to gather information about the signs and symptoms, and their duration experienced by the deceased, and events leading up to the death. The information collected is use to determine the individual COD using the International Classification of Diseases, Tenth Edition (ICD-10) [6]. The COD is assigned either directly by a trained physician or other automated methods. Verbal autopsy has been used as a valuable interim method to provide COD data, as countries work toward improving their civil and vital registration systems.

Despite the wide use of VA, there are many recognized limitations of VA tools and methods [7–11]. The World Health Organization (WHO) in recent years has commissioned systematic reviews of VA tools and methods and held technical consultation meetings, in an effort to update and standardize VA methods and tools to address some of these limitations, including comparability of VA data across study sites [10–13]. These reviews however, have had a more broad focus on how best to standardize tools and methods and have not thoroughly examined the specific limitations of VA methods and tools for measuring malaria mortality. Many VA studies have noted some of the specific limitations of VA tools and methods for measuring malaria mortality [14–32]; however, to date no systematic review has been conducted to examine the challenges and limitations of VA for measuring malaria mortality and to determine how VA methods could be improved to provide more robust estimates of malaria mortality. A systematic review of the literature was conducted to document how VA tools and approaches have been used to measure malaria mortality and the key challenges and limitations of existing tools and methods.

## Methods

The authors searched PubMed, the Cochrane Library, Popline, the WHOLIS, and Google Scholar, from 1 January 1990 to 15 January 2016. The search terms were “malaria” or “malaria mortality” and “cause specific mortality” and “verbal autopsy”/“post mortem interview”/“mortality surveillance”/“verbal post mortem.” We also searched available INDEPTH Network websites (27 websites from INDEPTH Network health and demographic surveillance system (HDSS) sites in Africa (21), Asia (5), and Oceana (1)) to identify additional programme reports, articles, and gray literature from the organization on malaria-specific mortality. References of included publications were also reviewed for other relevant studies. The inclusion criteria were publications that presented results from a VA study where malaria was an identified COD and/or publication that discussed limitations or challenges related to the measurement of malaria mortality through VA. The review was restricted to articles published in English.

Two authors independently searched the databases and websites. The titles and abstracts of the identified studies and reports were screened to determine if they met the inclusion criteria. Full texts of publications that passed the screening were reviewed to determine eligibility. A narrative synthesis of the publications that met the inclusion criteria was conducted. A narrative description was developed and information was extracted on key characteristics for each of the included publications using a standard matrix. Using this matrix, the reviewers carried out a thematic analysis of key challenges and limitations of measuring malaria mortality through VA.

## Results

### Overview of the inclusion strategies

**Fig. 1:**
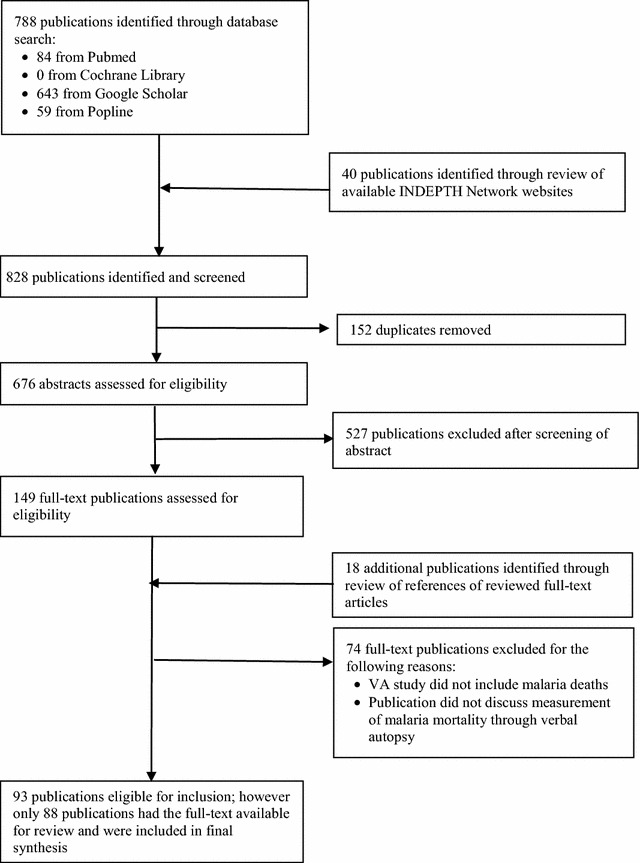
Flowchart showing the selection of publications on malaria mortality and verbal autopsy

The authors identified a total of 828 publications; 788 of these were identified through the database search and 40 through a review of the INDEPTH Network websites’ publications and reports. After removal of duplicates, the abstracts of 676 publications were reviewed for eligibility, and of these, 149 publications were selected for a full text review. In the full-text review of publications, 18 additional publications were identified through a review of references and 70 of the publications were excluded on the basis of non-inclusion of malaria deaths in the VA study or the publication did not discuss measurement of malaria mortality through VA. A total of 93 publications were included in the review; however, only 88 publications had the full text for review. Key information from the five publications not available for review is, therefore, unavailable and not included in the final synthesis (Fig. 1).

### Summary of articles included in the synthesis

Of the 88 publications included in the final review and synthesis, 64 were VA studies where malaria was included as a cause of death (one of which discussed the methods used in a VA validation study, but did not include results from the study). Of the 64 VA studies, 14 were VA validation studies and five were VA comparative methods studies. Nine of the publications were commentaries (4), systematic review or synthesis articles or reports discussing VA methods (4), and an editorial that discussed the issue of measurement of malaria mortality through VA. Eight of the articles presented global (4), regional (3), and country-level (1) malaria mortality estimates that drew from VA data and other sources to develop the estimates. The other publications included studies that used VA data/records to look at the effectiveness of interventions (4), the prevalence of care-seeking behavior prior to a malaria death (1), the impact of chloroquine resistance on malaria mortality (1); and the effect of misclassification bias in VA studies (1). The five publications that did not have the full text available included three VA studies and two studies that drew on VA data to assess the effectiveness of an intervention.

Key characteristics of the identified publications were extracted (Additional file 1), including the type of article; if it was a VA study or a study that used VA data, the setting/location of the study, the populations included, the sample size, the timeframe of the study, the VA instrument used in the study, the methods used for determining cause of death in the study, the reported sensitivity, specificity and positive predictive value in the study for malaria deaths, and information on or the criteria used to determine a malaria death in the study; and lastly, any challenges or limitations discussed related to the measurement of malaria mortality through verbal autopsy. Of the publications that were of VA studies, the majority were conducted in or included sites in Africa (60), while 14 of the studies included sites in Asia and 5 included sites in Central America (Mexico). Thirty of the VA studies included populations of all ages, 15 studies were specifically on children under 5 years only, seven included adults only (15 years and above), and one included only pregnant women ages 15–49. The remaining included either a specific age group or a combination of a few specific age groups. The most common method used to code the COD in VA studies was physician review (22), followed by Interpreting Verbal Autopsy Version 4 (InterVA-4) (12), while 30 studies used a combination of methods to code the COD (20). The majority of VA studies (51 of 64) did not include any information on how a malaria death was coded or the criteria used to code a malaria death; only 16 of the studies included detailed information on this, with the definition varying considerably across the studies. Of the 88 publications, 60 of them included discussions on limitations and challenges related to the measurement of malaria mortality through VA.

### Varying and low levels of sensitivity and specificity of malaria verbal autopsy tools

The most commonly cited limitation of measuring malaria mortality through VA was the varying and overall low levels of sensitivity and specificity of VA tools for measuring malaria mortality [3, 14, 16–21, 23, 24, 30–43]. This is confirmed through the results from the VA validation studies; with sensitivity ranging from 19 to 75% and specificity ranging from 69 to 100%. Several reasons were noted in the literature for the low and varying sensitivity and specificity. Verbal autopsy performs well for CODs that have a distinct set of signs and symptoms such as measles and malnutrition [21]. However, for malaria, it presents symptoms that overlap with other common CODs, including acute respiratory infections (ARI) and meningitis [43–52], which can result in misclassification bias. In areas with high HIV prevalence, it was also noted that it is more difficult to accurately attribute COD to malaria or HIV due to the overlap in symptoms [15, 41, 53].

The malaria epidemiological context also influences misclassification bias, resulting in either an under or over estimation of the malaria mortality burden [14, 16, 26, 31, 43, 48, 49, 54, 55]. In high transmission areas, it was suggested that malaria mortality is overestimated due to the practice of assigning malaria as the COD for cases of acute febrile illness where no other cause is evident [14]. Many studies have attributed this as bias introduced by the experience and knowledge of the physicians coding the deaths [7, 39, 45, 47–49, 52, 56–58]. However, a few studies note the reverse finding, that the malaria mortality burden is actually underestimated in high transmission areas and overestimated in medium to low malaria prevalence areas [26, 37]. In areas where malaria is highly seasonal, this also influences the COD determination and can lead to classification bias with malaria deaths more commonly classified during the peak transmission season [46, 52, 59]. Seasonality of other diseases that have overlapping symptoms with malaria, such as meningitis, can further influence COD determination and result in classification bias [46]. Another reason noted for the varying sensitivity and specificity includes the difficulty of assigning malaria as the underlying or as an indirect cause of death, where other factors could have contributed to the death [14, 35, 53, 60, 61]. For example, a few studies noted the difficulty in distinguishing between anaemia and malaria deaths [35, 53, 60], and the under recording of anaemia deaths as a result [35, 53]. Lastly, the availability of medical information, and more specifically, information on confirmed malaria through testing is often not available in VA data [57, 62, 63].

### Lack of comparability of verbal autopsy malaria mortality findings across sites

The lack of standardization in the application of VA tools and methods was another commonly cited limitation [14, 16, 18, 36, 41, 56, 64–67]. This includes differences in the format and content captured in VA questionnaires used across studies, and the specific age groups for which the questionnaires are designed [16, 64]. There are wide variations in the implementation of VA studies, including in the training provided to physicians, the type of interviewers used, respondent selection procedures, and the length of the recall period for capturing deaths [16, 52, 67]. The list of CODs and the specific coding or algorithms used to assign deaths, and specifically malaria deaths, varies substantially across VA studies [16]. VA studies also differ on whether one or more cause of death is assigned for each case and whether the narrative or open-ended section of the questionnaire is used to determine the cause of death [16, 67]. The majority of VA studies examined in this review provided limited information on the tools and methods used in the study, and specific details on how a malaria death was coded.

### Inadequacy of a gold standard in comparing malaria verbal autopsy study results

Another common limitation noted is the use of hospital medical records as the gold standard for which to compare VA study results. Hospital records in the settings in which VA studies take place often have data quality challenges [36, 42, 65] and typically reflect a different population than those of community-based VA studies [16, 28, 34, 36, 47, 58, 65, 68–71]. This is particularly so for malaria, where the majority of malaria deaths occur outside of the formal health system and lack information on confirmed malaria diagnosis [63].

### General limitations of verbal autopsy

Other general limitations of VA studies that were noted include the small sample sizes of studies that limit the precision of estimates [16, 19], recall bias among respondents that can result in misclassification bias [35], and the fact that many deaths in VA studies cannot be determined and are either excluded from the study (due to incomplete information) or are classified as an ‘unknown’ cause of death [39, 49, 72, 73].

## Discussion

Over the past few decades VA has been increasingly used as a valuable interim measure to provide data on mortality rates and the main COD in low- and middle-income countries, where civil and vital registration systems are incomplete and lacking quality data on mortality. Thus, filling a large gap in providing essential information for effective health policy and programmatic planning, particularly in contexts where resources are very limited. VA has been widely used to measure malaria-specific mortality, particularly in sub-Saharan Africa (SSA) where the greatest burden of the disease exists and where there is the largest gap in COD data. Although it is widely used, it is generally recognized in VA studies and by the global malaria community that VA does not perform particularly well, regardless of the COD assignment methods used, for determining malaria mortality. Though, to date, no collaborative efforts have been made to thoroughly examine and address the main challenges and limitations of VA for measuring malaria mortality.

This synthesis of the literature revealed the main limitation of VA for malaria mortality measurement to be its overall low and varying sensitivity and specificity, the reasons for which are multifaceted. The nonspecific symptoms of malaria make it difficult to distinguish malaria deaths from other common illnesses, most notably acute febrile illnesses such as ARI and meningitis, thus introducing misclassification bias. Due to this, in a few VA studies reviewed, malaria deaths were lumped under the category of ‘fever’ or ‘acute febrile illness’ death [51, 74–76]. The underlying COD profile also influences the sensitivity and specificity. A few studies for example noted the difficulty of distinguishing malaria from HIV deaths in high HIV prevalence areas due to the overlapping symptoms, suggesting that in these contexts malaria deaths are likely to be overestimated, while HIV-related deaths are underestimated [15, 41, 53]. Due to the complex aetiology of malaria, it can also be difficult to identify whether malaria was the direct or an underlying COD, or an indirect COD. A few studies noted the difficulty in distinguishing between a malaria and anaemia death [60, 61]; in the study by Murray et al. the authors note that they redistributed a proportion of anaemia deaths to malaria deaths due to this reason. Malaria infection has also been shown to associated with an increased risk for potentially fatal invasive bacterial infections, including non-typhoidal Salmonellae [77]; in these cases, malaria will not be recorded as the COD despite its role in indirectly influencing the death. The malaria epidemiological context, including areas where malaria is highly seasonal, also influences the sensitivity and specificity of VA for malaria mortality, with potential bias introduced by physicians’ backgrounds and experience [39, 42, 45, 47–49, 52, 56–58, 61, 62]; resulting in either an under or over estimation of the malaria mortality burden.

The lack of standardization of tools and methods used in VA studies is another key challenge, as it makes comparability of malaria mortality findings across sites and over time difficult [11, 14, 36, 56, 64, 78]. This challenge is further exacerbated by the lack of detailed information provided in published studies on the tools and methods used, which was evident in this review. While the lack of standardized tools and methods is a recognized overarching issue in general for VA studies, it further complicates being able to assess from the literature what the best practices are for measuring malaria mortality through VA. For example, most VA studies in the review did not provide information on the criteria used to assign a malaria COD and for the studies that did include this information, the criteria used ranged widely across studies; thus providing little insight on the most accurate cause of death assignment for a malaria death. On the other hand, VA can still be a valuable tool for monitoring and evaluating trends in malaria mortality in specific settings over time when the same methods are applied consistently, as they are affected by the same set of biases over time [56, 79].

A number of the studies also discussed the challenge of not having a ‘true’ gold standard by which to test the performance of VA and, therefore, caution in the interpretation of validation study findings [16, 28, 34, 36, 42, 47, 58, 65, 68, 69, 71]. For malaria specifically, it is not just a challenge of incomplete and poor quality records, but in many validation sites the coverage of parasitological testing is incomplete and therefore, the malaria COD diagnosis is made without a confirmed laboratory test. The population health metrics research consortium (PHMRC) gold standard VA validation study initiated in 2005 is helping to address this issue through the development and use of stringent diagnostic criteria to identify gold standard deaths, thus providing a better understanding for VA performance for malaria mortality measurement across different COD assignment methods and guidance for future validation studies [65].

To provide more robust malaria mortality estimates moving forward, it is pertinent that the global malaria community come together to review the limitations of malaria mortality data sources and methods and define a strategy for how these methods could be improved upon. Given the significant contribution of VA data in informing global malaria mortality estimates, it will be important for the strategy to include revisiting current VA tools and methods for measuring malaria mortality to determine if updates could provide improved estimates. This could be an opportunity for the field to better refine and improve the criteria used for assigning malaria deaths in VA studies and ultimately improve the sensitivity and specificity of current VA tools. There is also a strong need for better collaboration across VA experts and transparency of methods used in VA studies to ensure better standardization of VA methods and to allow for greater comparability across study findings. Further, in view of the increasing coverage of parasitological testing in malaria endemic countries, it is possible, as shown in the PMHRC validation study, to use more stringent diagnostic criteria for assigning malaria deaths.

Lessons learned from this study should be used to inform future VA validation studies. It is also important we continue to explore new strategies. Very recently, minimally invasive tissue sampling (MITS) for autopsy has emerged as a potential new method for determining COD in developing countries where full autopsies are not possible [80–82]. This technique offers the potential for improved diagnostic accuracy of COD, and could potentially in the long term obviate the need for VA studies. Exploration and development of these new strategies should happen alongside longer-term efforts to improve civil and vital registration systems in low- and middle-income countries.

## Conclusions

This review sheds light on the key limitations for measuring malaria mortality through VA. It also highlights the need for the global malaria community to come together to define a strategy for improving current methods for more robust measurement of malaria mortality in the future, an effort that should include exploring whether VA tools and methods can be improved. This will be pertinent to measure progress toward the ambitious malaria control and elimination goals set forth in the Sustainable Development Goals and the *Global Technical Strategy for Malaria 2016*–*2030.* Longer-term strategies should focus on improving countries’ vital registration systems for more accurate and timely cause of death data.

## Additional file

**Additional file 1.** Summary of selected publications on malaria mortality and verbal autopsy.
